# Identify potential prognostic indicators and tumor-infiltrating immune cells in pancreatic adenocarcinoma

**DOI:** 10.1042/BSR20212523

**Published:** 2022-02-18

**Authors:** Ting Shi, Ge Gao

**Affiliations:** Department of Clinical Laboratory, Hunan Provincial People’s Hospital (The First-Affiliated Hospital of Hunan Normal University), Changsha 410005, Hunan, China

**Keywords:** pancreatic adenocarcinoma, tumor microenvironment, tumor-infiltrating immune cells

## Abstract

**Background**: Pancreatic adenocarcinoma (PAAD) is a kind of highly malignant tumor and lacks early diagnosis method and effective treatment. Tumor microenvironment (TME) is of great importance for the occurrence and development of PAAD. Thus, a comprehensive overview of genes and tumor-infiltrating immune cells (TICs) related to TME dynamic changes conduce to develop novel therapeutic targets and prognostic indicators.

**Methods**: We used MAlignant Tumors using Expression data (ESTIMATE) algorithm to analyze the transcriptome RNA-seq data of 182 PAAD cases on The Cancer Genome Atlas (TCGA) platform. Gene Ontology (GO), Kyoto Encyclopedia of Genes and Genomes (KEGG), protein–protein interaction (PPI) network, COX regression analysis and gene set enrichment analysis (GSEA) were carried out to get the hub genes related to the prognosis of PAAD patients. These core genes were validated in GEPIA. CXCL10 expression as a poor prognostic indicator was validated in GEO database. Finally, CIBERSORT algorithm was applied to understand the status of TICs.

**Results**: A total of 715 up-regulated differential expression genes (DEGs) and 57 down-regulated DEGs were found simultaneously in stromal and immune groups. These DEGs were mainly enriched in immune recognition, activation and response processes. CD4, CXCL12, CXCL10, CCL5 and CXCL9 were the top five core genes. Then, the validation of these genes showed that CD4, CXCL10, CXCL5, CXCL9 were up-regulated in PAAD. Among the core genes, CXCL10 had a negative correlation with the survival time of PAAD patients. CD8+ T cells, CD4+ T cells memory activated, macrophages M1 had positive correlation of CXCL10 expression, whereas regulatory T cells (Tregs), macrophages M0 and B cells memory had negative correlation.

**Conclusion**: We generated a series of genes related to TME with prognostic implications and TICs in PAAD, which have the potential to be novel immunotherapy targets and prognostic markers. The data showed that CXCL10 was favorable as a poor prognostic indicator in PAAD patients.

## Introduction

Pancreatic adenocarcinoma (PAAD), a highly malignant gastrointestinal tumor, has a poor prognosis with a 5-year survival rate of 2–9%. PAAD is the seventh leading cause of cancer deaths in industrialized countries [1]. Because the early symptoms of PAAD are not obvious and lack of effective diagnosis method, 80% of diagnosed patients are in the middle or advanced stage of the cancer. What has been worse is that surgical resection is not available for the patients with advanced stage and distant metastasis [2]. PAAD also has low sensitivity or high resistance to radiotherapy and chemotherapy so that it is difficult to obtain a good prognosis [3]. Therefore, it is of great significance to find new therapeutic methods and diagnostic indicators for PAAD.

Recently, an increasing number of studies demonstrated the occurrence and development of tumors are not only related to tumor cells, but also are regulated by tumor microenvironment (TME) [4–6]. Vascular endothelial cell, mesenchymal stem cell, tumor-associated fibroblast and immune cell are the cellular components (CCs) of TME [7]. Once the TME is formed, a large number of immune cells such as tumor-infiltrating lymphocytes (TILs), tumor-associated macrophages (TAMs), regulatory T cells (Tregs), dendritic cells etc., will be recruited to tumor site [8]. Immune cells and stromal cells as two leading components of TME mediate various processes of tumor formation and growth by inducing angiogenesis, evasion of immune surveillance, inhibiting cell apoptosis, sustaining proliferation and promoting epithelial–mesenchymal transition [9]. In addition to CCs, TME also contains extracellular matrix molecules. Interleukins, growth factors, matrix metalloproteinases and chemokines along with immunosuppressive cells in the TME jointly promote tumor immune escape, tumor growth and metastasis [10–12]. The cells and molecules in TME are in a dynamic change process which reflects the essence of TME evolution. Moreover, TME impacts the effect of immunotherapy in which the components have potential to be prognostic markers. So comprehensive overview of dynamic molecular changes and the infiltration status of non-tumor cells may improve our understanding of molecular mechanism in tumor process and conduce to develop novel therapeutic targets and prognostic indicators. According to specific molecular signature expression of immune and stromal cells, Yoshihara et al. described a MAlignant Tumors using Expression data (ESTIMATE) algorithm which applied ImmuneScore and StromalScore to evaluate the level of infiltrating immune and stromal cells in tumor samples, and used EstimateScore to assess the purity of tumor [13]. Based on the description above, researchers have obtained overall picture of TME and have investigated assessment of prognostic indicator and exploration of molecular alterations in many tumors, such as clear cell renal cell carcinoma [14], glioblastoma [15], breast cancer [16], lung adenocarcinoma [17]. However, the application of the ESTIMATE algorithm and its corresponding effect in PAAD has not been fully investigated to the best of the authors’ knowledge.

Because of the important roles of tumor-infiltrating immune cells (TICs) and other stromal components in PAAD, we first used ESTIMATE algorithm to analyze the transcriptome RNA-seq data of 182 PAAD cases on The Cancer Genome Atlas (TCGA) platform. Then the differential expression genes (DEGs) related to TME were obtained. Gene Ontology (GO), Kyoto Encyclopedia of Genes and Genomes (KEGG), protein–protein interaction (PPI) network and survival analysis were all carried out to get the hub genes related to prognosis of PAAD patients. Moreover, these core genes were validated in GEPIA. CXCL10 expression as a poor prognostic indicator was validated in GEO database. Finally, CIBERSORT algorithm was applied to illustrate the correlation of hub gene and TICs.

## Methods

### Raw data

We downloaded the transcriptome RNA-seq data of 182 PAAD cases and the corresponding clinical data such as age, gender, grade, stage, TMN staging and survival time from TCGA platform (https://portal.gdc.cancer.gov/). In order to verify the precision of gene expression, we downloaded the GSE28735, GSE62452, GSE28735, GSE71729, GSE11838 datasets from NCBI (https://www.ncbi.nlm.nih.gov/gds/) for further analysis.

### Calculation of the stromal/immune/estimate score

ESTIMATE algorithm analyzed specific gene expression to evaluate immune and stromal infiltration in TME. We installed ‘estimate’ R package in R 4.0.2 (https://www.r-project.org/). Each sample can obtain ImmuneScore and StromalScore. ImmuneScore and StromalScore were positively correlated with the ratio of immune and stromal cells, respectively. ESTIMATEScore is the summation of ImmuneScore and StromalScore which can predict the purity of tumor.

### Screening of DEGs

According to the medians of ImmuneScore and StromalScore (the median in stromal group was 770.40 and in immune group was 1158.75), 178 PAAD samples were divided into high- and low-score groups, respectively. Wilcox test was used to analyze the differential gene expression. DEGs were screened out between high- and low-score groups with the screening criterion FDR < 0.05 and |logFC| > 1. The screened DEGs were visualized through heatmap via R-package, pheatmap. In order to further explore the hub genes, the Venn graphs were applied to display the intersection DEGs that were up- or down-regulated synchronously in immune and stromal groups.

### Enrichment analysis and PPI network

GO and KEGG analyses were performed for 772 intersection DEGs with enrichment *P*<0.05 and *q*<0.05.

The PPI network was constructed on Search Tool for the Retrieval of Interacting Genes (STRING) website. Setting the minimum required interaction score to the confidence level of 0.95. And then the PPI network was reconstructed by Cytoscape software to elucidate the potential interactions of DEGs and identify the core genes which have strong connection in network.

### COX regression analysis and survival analysis of hub gene

We installed ‘survival’ and ‘survminer’ package in R and then used it for univariate COX regression and survival analysis. The top 16 genes related to prognosis of PAAD patients ordered by *P*-value were shown in forest plot. Among these 16 genes and the core genes in PPI network, the intersection gene was selected to do survival analysis. Kaplan–Meier analysis was used to drawn survival curve.

### Verification of core genes in GEPIA

GEPIA is an interactive web tool for gene differential expression analysis. GEPIA can analyze pancreatic cancer data with convenience. We simulated the different expression of the core genes between 179 primary pancreatic cancer samples and 171 normal samples by using GEPIA. *P*<0.05 was judged to be statistically significant.

### Gene set enrichment analysis

We loaded hub gene data on gene set enrichment analysis (GSEA) 4.1.0. Selected hallmark gene set v7.1, c2 kegg gene set v7.1 and c7 immunologic signatures gene set v7.1 to do the enrichment analysis. Only the gene set with FDR *q*<0.05 and NOM *P*<0.05 were considered statistically significant.

### Correlation analysis of hub gene and TICs

Using CIBERSORT algorithm in R to get the proportion of TICs in each sample. Subsequently, according to the median value of the hub gene, the 178 tumor samples were divided into high- and low-expression groups, using ‘limma,’ ‘ggpubr,’ ‘gglot2,’ ‘vioplot,’ and ‘ggExtra’ packages in R language for difference analysis and correlation analysis of TICs.

## Results

### Screening of intersection DEGs based on ImmuneScore and StromalScore

**Figure 1:**
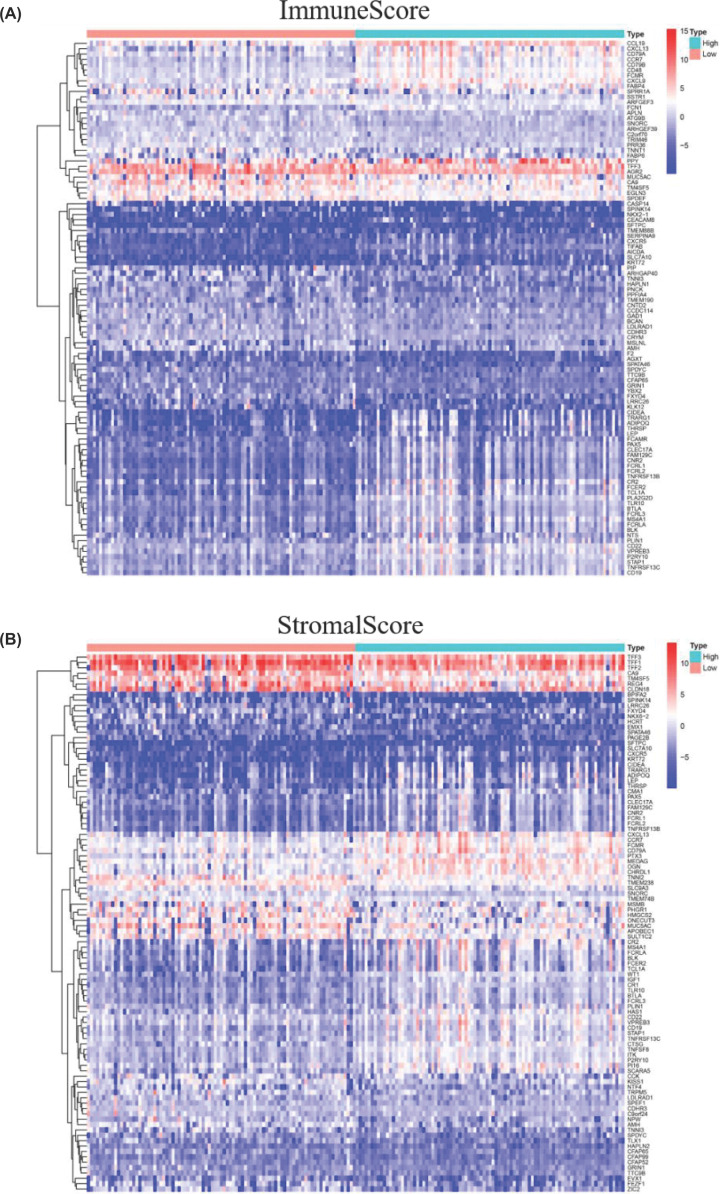
Heatmap graph for DEGs (**A**) Heatmap for DEGs based on ImmuneScore. (**B**) Heatmap for DEGs based on StromalScore (high-score groups compared with low-score groups). The horizontal axis represented 178 PAAD samples and the vertical axis represented the names of DEGs.

**Figure 2:**
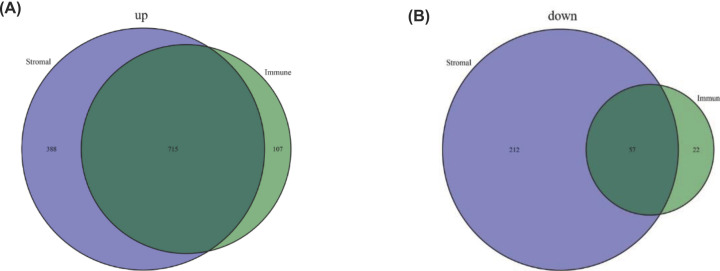
Venn graph for DEGs (**A**) Venn graph showed the intersection DEGs that are up-regulated synchronously in stromal and immune groups. (**B**) Venn graph showed the intersection DEGs that are down-regulated synchronously in stromal and immune groups.

To explore the relationship between the alterations of gene expression profile and immune/stromal component in TME, we investigated differential gene expression analysis between high/low score in both immune and stromal score group. A total of 901 DEGs were found in high/low ImmuneScore group including 822 up-regulated DEGs and 79 down-regulated DEGs (Figure 1A). Moreover, 1372 DEGs were obtained in high/low StromalScore group including 1103 up-regulated DEGs and 269 down-regulated DEGs (Figure 1B). To reduce the system error caused by group classification, the intersection DEGs, up- or down-regulated synchronously in stromal and immune groups were deemed as valid genes. Finally, 715 identical up-regulated DEGs and 57 identical down-regulated DEGs were found and the results presented by Venn graph (Figure 2A,B).

### GO and KEGG analyses of valid DEGs

**Figure 3:**
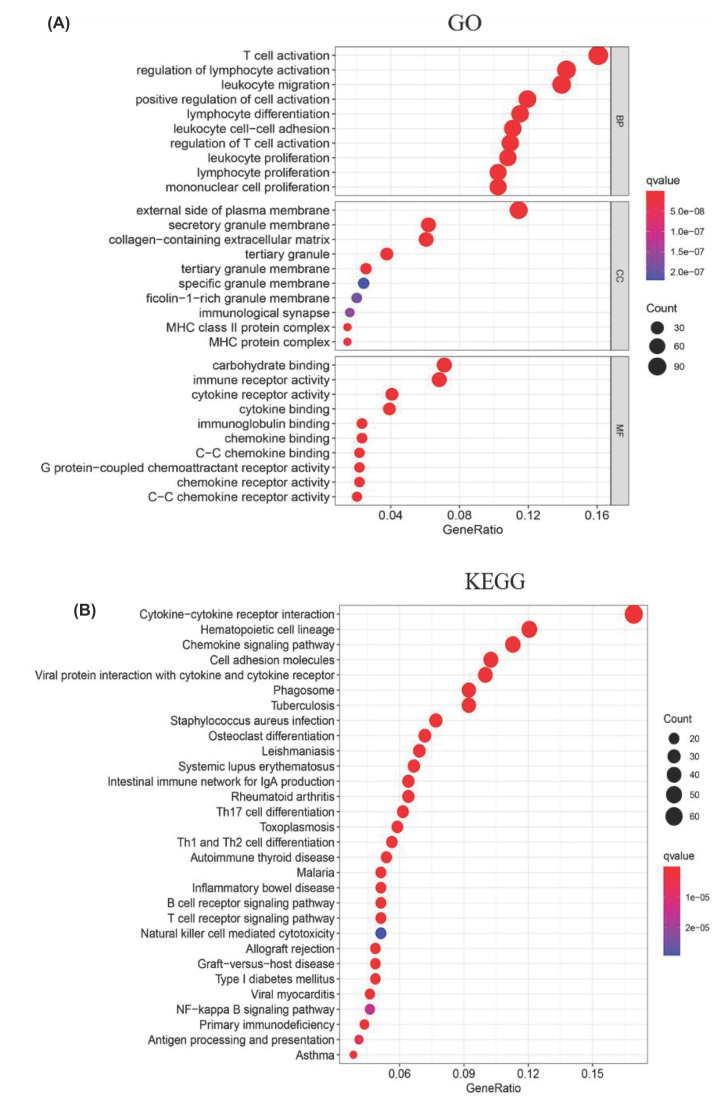
GO and KEGG analyses for DEGs (**A**) Top 30 GO terms of DEGs. (**B**) Top 30 KEGG pathways of DEGs. The color of nodes indicated *q* value and the size of nodes showed the count of DEGs enriched.

**Figure 4:**
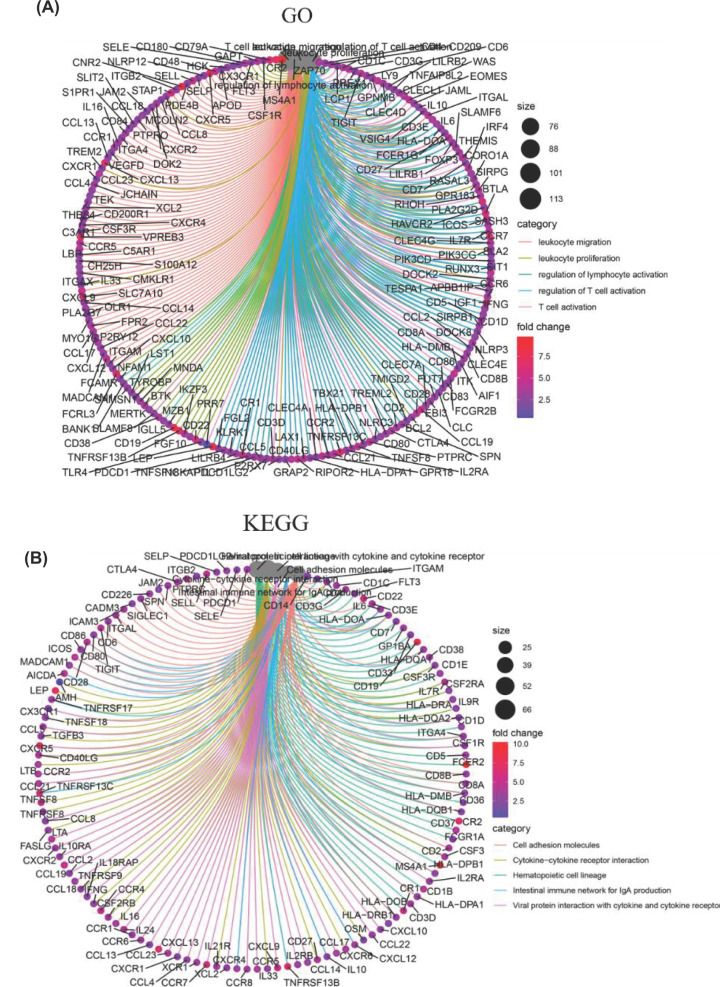
GO and KEGG analysis for DEGs (**A**) The cycle graph of DEGs enriched in the top five GO terms. (**B**) The cycle graph of DEGs enriched in the top five KEGG pathways. Each dot represented a DEG and different colors of lines meant different GO terms and KEGG pathways.

In order to understand the gene function and the pathway of valid DEGs involved, we performed GO and KEGG analyses of 772 DEGs. The top ten terms of biological processes (BPs), CCs and molecular functions (MFs) were listed separately (Figure 3A). In BPs, GO functions were mainly enriched in immune cells activation, proliferation and migration. CCs regarding membrane, extracellular matrix and antigen presentation complex were identified. MFs including cytokine, immunoglobulin and chemokine binding and its receptor activity were found. Similarly, the pathways related to immune processes and regulations were also found in KEGG enrichment analysis, such as B, T cell receptor signaling pathway, cytokine–cytokine receptor interaction etc. (Figure 3B). These results suggested that immune factors were critical in TME of PAAD. Besides, the DEGs enriched in the top five BPs and KEGG pathways were displayed in Figure 4A,B.

### Obtaining the hub gene via PPI network analysis and univariate COX regression analysis

**Figure 5:**
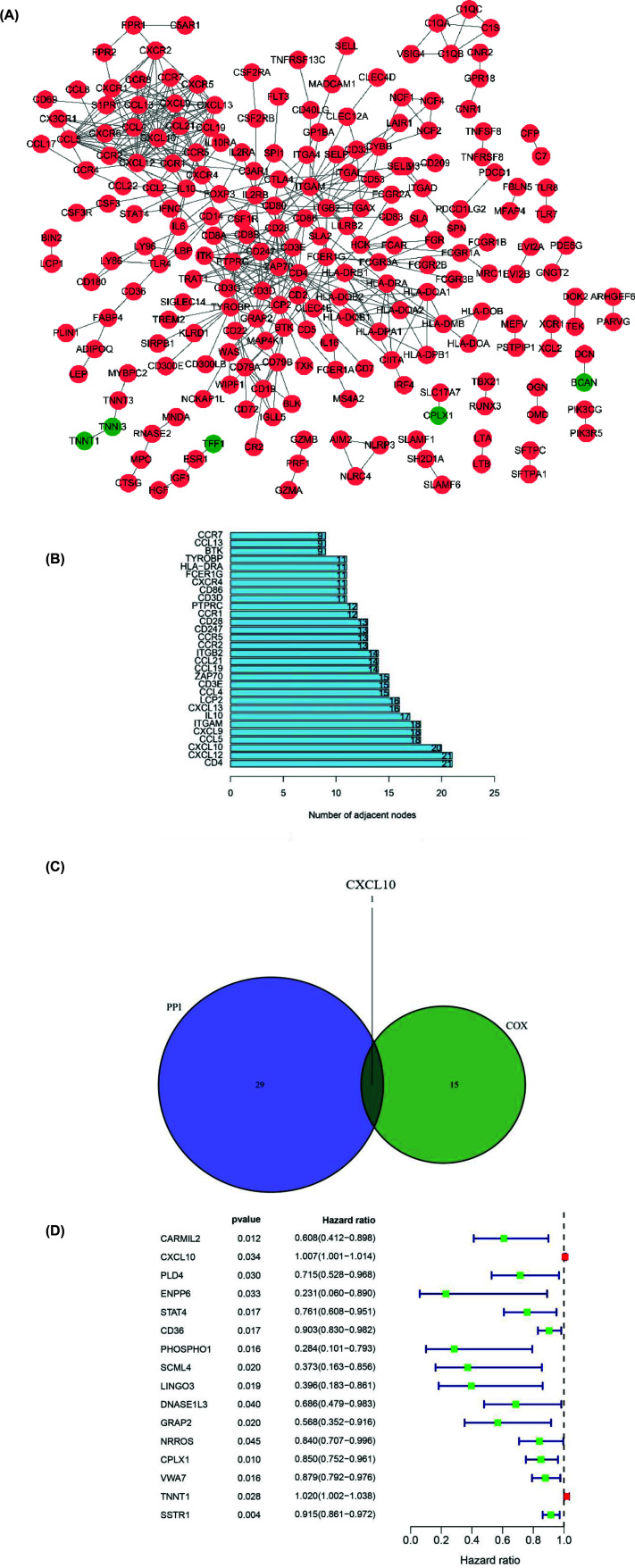
PPI network and univariate COX regression analysis (**A**) The interaction network of proteins encoded by DEGs. Red dots meant up-regulated genes, whereas green dots meant down-regulated genes. (**B**) The top 30 core gene in PPI network. (**C**) Venn graph showed the intersection DEG was not only the core gene in PPI network but also prognosis-related genes. (**D**) Sixteen DEGs related to the prognosis of PAAD patients.

**Figure 6:**
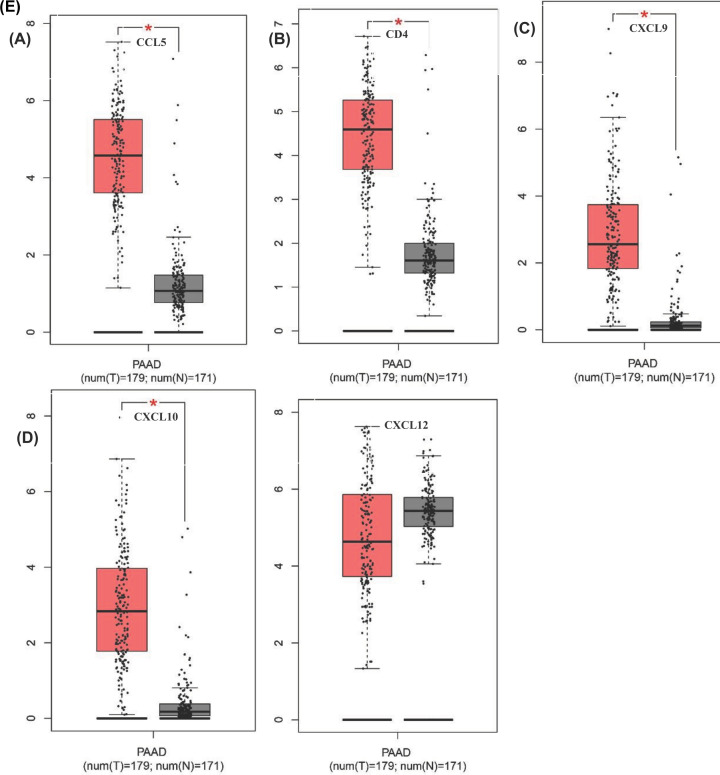
The expression level of mRNA of hub gene in 179 PAAD samples and 171 normal samples (**A**) CCL5. (**B**) CD4. (**C**) CXCL9. (**D**) CXCL10. (**E**) CXCL12. T represented primary PAAD samples. N represented normal samples. * represented T *vs* N and *P*<0.05 was considered statistically significant.

PPI network was constructed to elucidate the relationships among DEGs (Figure 5A) and the core genes in PPI network were listed in bar plots (Figure 5B). CD4, CXCL12, CXCL10, CCL5 and CXCL9 were the top five core genes. We used GEPIA to verify the accuracy of the results. CD4, CXCL10, CCL5 and CXCL9 were up-regulated in PAAD, whereas there was no significant difference in CXCL12 expression (Figure 6). Univariate COX regression analysis showed that 16 DEGs were associated with the prognosis of PAAD patients (Figure 5D). In order to find out the core genes related to the prognosis, we did the intersection analysis of 30 core genes in PPI network and 16 prognosis-associated genes. Ultimately, only one gene, CXCL10, was identified (Figure 5C).

### Survival analysis and GSEA of CXCL10

**Figure 7:**
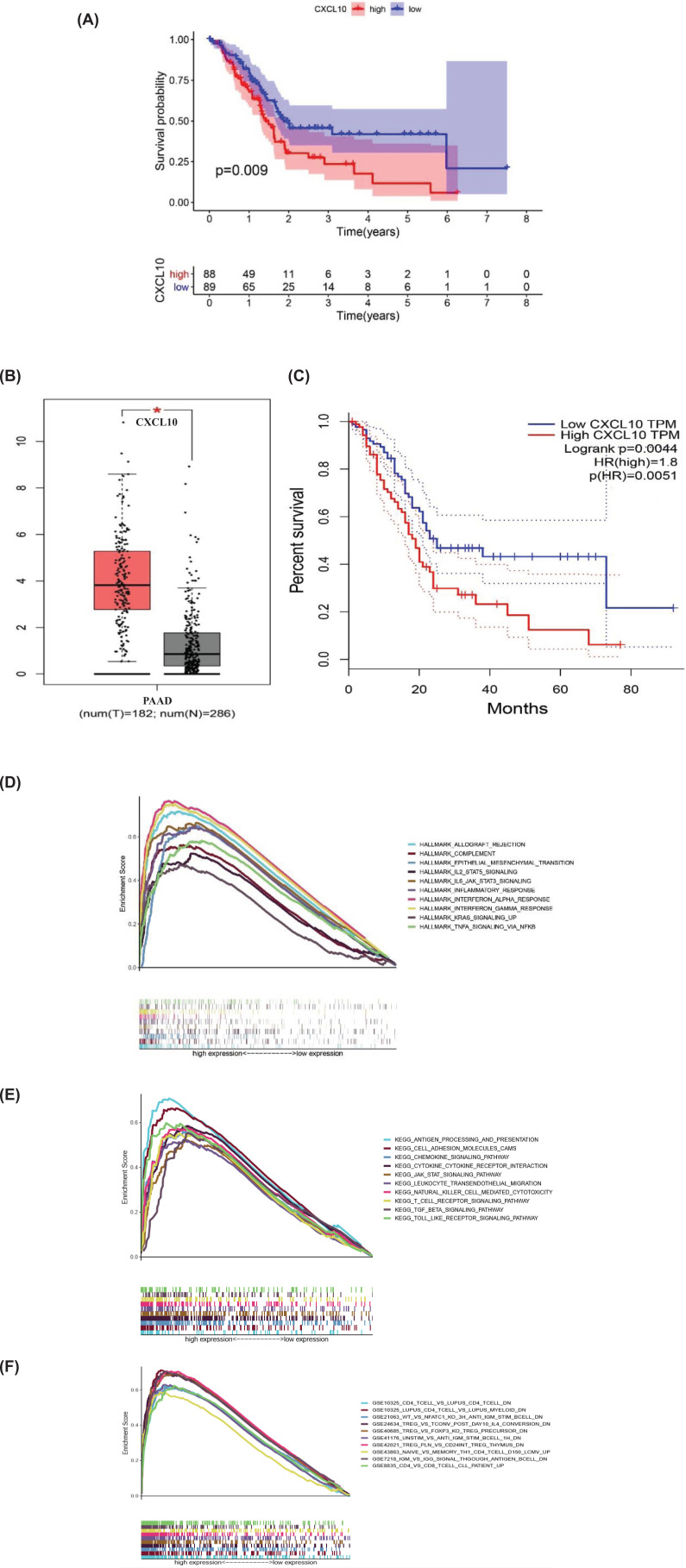
Survival analysis and gene-set enrichment analysis of CXCL10 (**A**) Survival analysis for PAAD patient with high and low expression of CXCL10. (**B**) Validation of CXCL10 expression in GSE28735, GSE62452, GSE28735, GSE71729, GSE11838 datasets. (**C**) Survival analysis of CXCL10 for PAAD in GSE28735, GSE62452, GSE28735, GSE71729, GSE11838 datasets. (**D**) The GSEA graph for hallmark gene set, each line of different color represented a statistically significant gene set (FDR *q*<0.05 and NOM *P*<0.05). The horizontal axis represented genes and the left and right sides of the horizontal axis indicated high- and low-expression genes, respectively. (**E**) The GSEA graph for KEGG gene set. (**F**) The GSEA graph for c7 immunologic signatures gene set.

According to the median of CXCL10 expression level, we divided 178 PAAD samples into high- and low-expression level group of CXCL10. Then we did the survival analysis. The result indicated that the survival time of patients with high expression of CXCL10 was shorter than that of patients with low expression (Figure 7A). The expression of CXCL10 had a negative correlation with the survival time of patients. In GEO database, we validated that CXCL10 was highly expressed in cancer cases and negatively correlated with the prognosis of PAAD (Figure 7B,C).

To further explore the potential mechanism of CXCL10 affecting tumor occurrence and development in TME, three gene sets were selected for GSEA of high- and low-expression groups. For hallmark gene set, genes in high CXCL10 expression group were mainly enriched in immune molecule-related processes such as inflammatory response, interferon response, complement and interleukin signaling (Figure 7D). For KEGG gene set, antigen processing and presentation, cell adhesion molecules, chemokine signaling pathway and other processes were enriched, indicating the underlying mechanism and pathways activated in TME related to tumor progression (Figure 7E). Moreover, we also followed the interest in c7 immunologic signatures gene set. Immune cell such as CD4^+^ T cell, B cell, T cell regulatory were enriched (Figure 7F). All these results suggested CXCL10 may be an important factor in regulating the dynamic changes in TME.

### Correlation of CXCL10 with the proportion TICs

**Figure 8:**
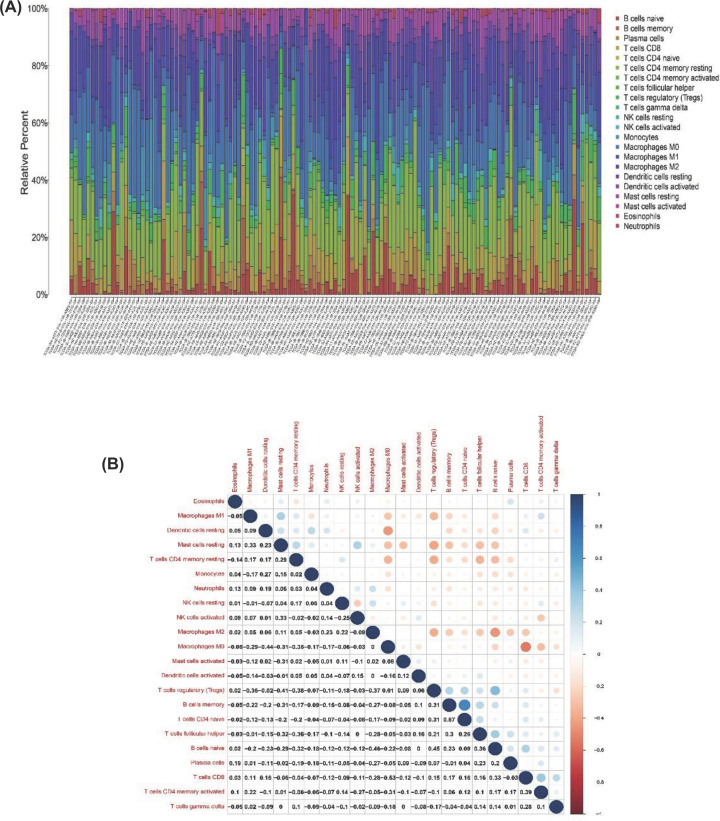
The analyzed data of 22 kinds of TICs (**A**) Bar graph displayed the proportion of 22 kinds of TICs in TME of 178 PAAD patients. The horizontal axis represented 178 tumor samples. The vertical axis indicated the proportion of TICs, different color represented different immune cells. (**B**) The graph showed the correlation between immune cells. The blue circle represented a positive correlation, whereas the red circle represented a negative correlation.

**Figure 9:**
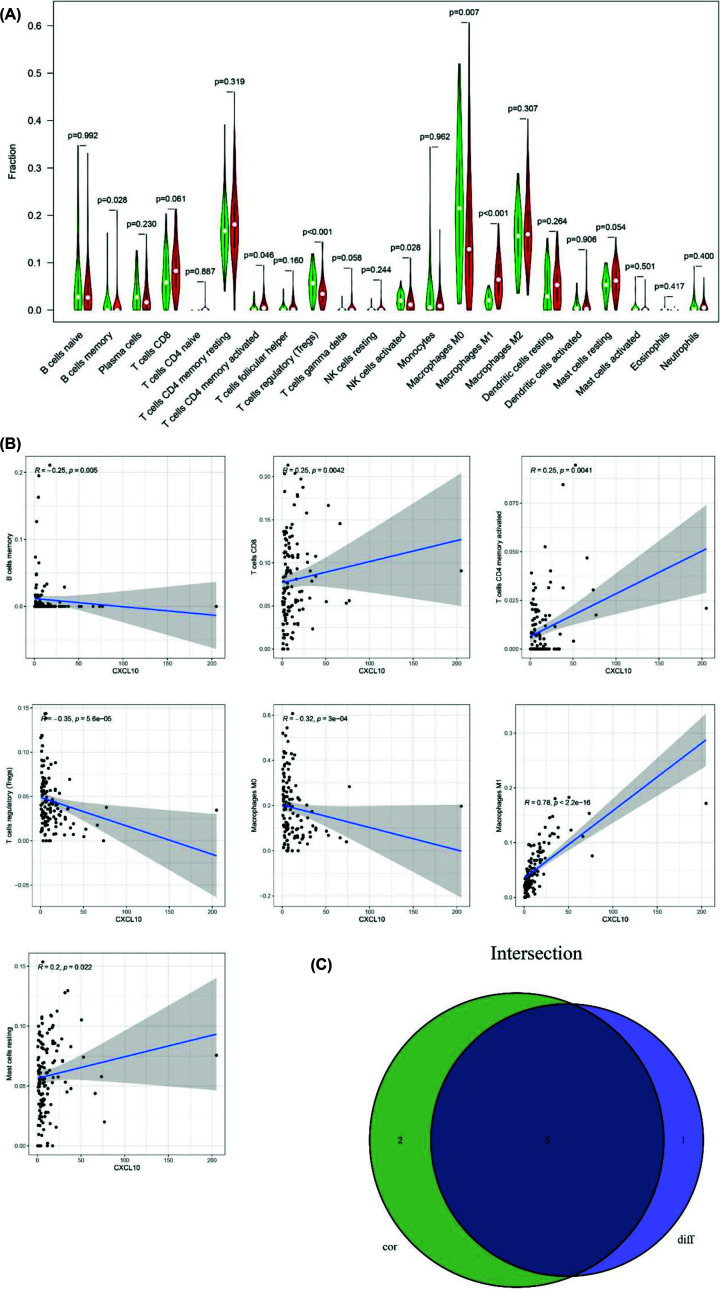
The relationship between CXCL 10 and TICs (**A**) The differential analysis of 22 kinds of TICs in high and low CXCL10 expression groups. Green represented low-expression group and red represented high-expression group. (**B**) Correlation analysis between CXCL10 expression and seven kinds of immune cells. (**C**) Venn graph showed the intersection TIC of differential and correlation analysis.

We used the CIBERSORT algorithm to analyze the proportion of 22 kinds of TICs in TME among 178 PAAD patients (Figure 8). Furthermore, difference analysis and correlation analysis of immune cells were performed to illustrate the correlation of CXCL10 with TICs. In difference analysis, six kinds of immune cells were found having statistical significance including B cell memory, T cells CD4 memory activated, T cells regulatory (Tregs), NK cells activated, macrophages M0 and macrophages M1 (Figure 9A). In correlation analysis, T cells CD8, T cells CD4 memory activated, macrophages M1 and mast cells resting all had positive correlation of CXCL10 expression, whereas T cells regulatory (Tregs), macrophages M0 and B cells memory had negative correlation (Figure 9B). Ultimately, Venn graph showed the overlapped TICs of difference and correlation analysis (Figure 9C).

## Discussion

The TME plays an important role in the occurrence and development of cancer. The overview of the dynamically changing molecules in TME and the state of immune infiltrating cells help to understand the molecular mechanism in tumor progression process and discover the new therapeutic targets. In our study, we investigated the differential gene expression analysis between high/low score in both immune and stromal score group. The intersection genes, including 715 up-regulated DEGs and 57 down-regulated DEGs were identified in immune and stromal groups. Following GO and KEGG functional enrichment analyses, the overall function of these DEGs is mainly mapped on regulating the infiltration and the state of TICs and mediating inflammatory response, complying with the previous studies’ results [18–20]. In PPI network, CD4, CXCL12, CXCL10, CCL5 and CXCL9 were the top five core genes. The verified results showed that CD4, CXCL10, CCL5 and CXCL9 were up-regulated in PAAD, whereas there was no significant difference in CXCL12 expression. Four of them were chemokines. Chemokines are low-molecular weight proteins secreted by cells [21]. They induce chemotaxis in cells by binding the specific G protein-coupled seven transmembrane domain receptors [22]. Recent studies have manifested that chemokines directly affect tumor cells or indirectly recruit the immune cells in TME to regulate tumor angiogenesis, promoting or inhibiting tumor growth, invasion and metastasis [23]. CXCL12 activates tumor cells and attracts Tregs and MDSCs by binding with its receptor CXCR4 to induce angiogenesis and create an immunosuppressive environment for tumor cell proliferation [24]. CCL5 is highly expressed in the serum of breast cancer and the patients with high expression of CCL5 were more likely to have lymph node metastasis [25]. CCL5 recruits the TAMs which possess the pro-tumor properties to suppress the anti-tumor immune response and produce vascular endothelial growth factor (VEGF), fibroblast growth factor (FGF) to promote angiogenesis, epithelial-to-mesenchymal transition [26]. CXCL9 and CXCL10 have dual roles in tumorigenesis. They possess antitumor properties by which they guide the CD8^+^ T cells into the tumor site to induce apoptosis of tumor cells, attract CD4^+^ T cells to reduce angiogenesis and suppress cell proliferation. However, they also contribute to tumor progression and metastasis [27].

Next, we did the intersection analysis of 30 core genes in the PPI network and 16 prognosis-associated genes. Ultimately, only one gene, CXCL10, was identified as the core gene related to prognosis. Hallmark, KEGG, and immunologic signatures gene sets were selected for GSEA in our study. We discovered that most of them were enriched in immune cells, immune molecules and immune-related signaling pathways, and the results were consistent with the previous literature that CXCL10 was involved in chronic inflammation, immune dysfunction and tumor development [28]. These results suggested CXCL10 may be an essential factor in regulating the dynamic changes of TME and has the potential to be a novel immunotherapy target. CXCL10 also known as IFN-γ inducible protein 10 (IP-10), was first discovered in U-937 cells when Luster et al. studied the immune response induced by IFN-γ [29]. CXCL10 is secreted by different types of cells such as leukocytes, monocytes, neutrophils, eosinophils, epithelial cells, endothelial cells, stromal cells and tumor cells. Lunardi et al. identified that pancreatic stellate cells in pancreatic ductal carcinoma stroma were the main source of CXCL10 [30]. CXCL10 appears to have dual effects on tumor progression which depends on the type of CXCR3 receptor [31]. CXCR3-A as the main isoforms favors cell proliferation. Overexpression of CXCR3-A and its ligand CXCL10 mediate p38/MAPK, ERK1/2, JNK signaling pathway to cause intracellular calcium influx and promote tumor invasion, migration and metastasis [28,32]. Whereas CXCR3-B possesses the opposite effects of CXCR3-A. The binding of CXCL10 with CXCR3-B can inhibit tumor cell proliferation, migration and suppress the immune responses [33]. Some researchers have found that the CXCL10 can be a poor prognostic indicator of renal cancer and breast cancer [34,35]. In our survival analysis of CXCL10, we also found that the expression of CXCL10 had a negative correlation with the survival time of PAAD patients.

The TME of PAAD is unique in that it contains a large number of matrix components and immune cells. Multiple studies have shown that the proportion and the function of TICs are closely related to the occurrence and development of PAAD. In the early stage of PAAD, M0 macrophages exhibit antitumor property by secreting TNF-α to induce the cell cycle arrest and apoptosis of cancer cells [36]. And this antitumor property will be diminished when the macrophages differentiate into M1 and M2 types. Macrophages M1, as the classical macrophages, have proinflammatory and antitumor properties, while macrophages M2 have protumor properties [37]. However, some researchers have found during early carcinogenesis M1 macrophages promote tumor initiation by chronic inflammation in TME [20]. M1 macrophages activated oncogenes and constructed inflammatory milieu to promote tumor cell proliferation and survival, as well as other processes including angiogenesis and thus inhibit immune response [38]. These could explain why the expression of CXCL10 was negatively correlated with M0 macrophages and positively correlated with M1 macrophages in our study. Based on the explanation, we speculated that the aberrant expression of CXCL10 may be involved in the early stage of PAAD initiation and progression by regulating the function and proportion of M0 and M1 macrophages. The specific mechanism needs to be further investigated.

Besides revealing macrophages, some researchers have found that CXCL10 activates its receptor CXCR3 to induce the infiltration of CD8+ T cells in colorectal cancer [39], breast cancer [40], melanoma [41]. CXCL10 is a chemoattractant for CD8+ T cells and Tregs isolated from the peripheral blood of PAAD patients [29]. Our results showed that CXCL10 was positively correlated with CD8+ T cells, which was consistent with these results. Tregs restrain anticancer immune response, create an immunosuppressive TME to promote tumor growth [19]. The prevalence of Tregs was verified as a poor prognostic factor and was associated with tumor differentiation of PAAD [42]. But in our study, we discovered CXCL10 was negatively correlated with Tregs. Previous study identified the distribution of Tregs in TME change dynamically due to Tregs was abundant at day 7 but decreased with tumor progression [43]. Thus, the dynamic changes of CXCL10 in cancer progression and its relationship with Tregs during this change need to be further studied.

In summary, ESTIMATE algorithm was used to analyze the proportion of immune cells and stromal cells in 182 PAAD cases from TCGA platform. We generated a series of genes related to TME. CD4, CXCL12, CXCL10, CCL5 and CXCL9 were the top five core genes. CXCL10 was the core gene that had a negative correlation with the survival time of the patients, thus it can be a poor prognostic indicator of PAAD. Moreover, the expression of CXCL10 correlated with TILs. Whether the combination of these genes predict the prognosis better than a single gene and whether the gene can be important factors in regulating the dynamic changes of TME and novel immunotherapy target should be further investigated.

## Data Availability

All data are available from the open-access repository, MetaboLights. The transcriptome RNA-seq data of 182 PAAD cases and the corresponding clinical data such as age, gender, grade, stage, TMN staging and survival time are available from TCGA platform (https://portal.gdc.cancer.gov/). The GSE28735, GSE62452, GSE28735, GSE71729, and GSE11838 datasets were downloaded from NCBI (https://www.ncbi.nlm.nih.gov/gds/).

## Abbreviations

BP: biological process
CC: cellular component
DEG: differential expression gene
ESTIMATE: Estimation of Stromal and Immune cells in MAlignant Tumors using Expression data
GO: Gene Ontology
GSEA: gene set enrichment analysis
KEGG: Kyoto Encyclopedia of Genes and Genomes
MF: molecular function
PAAD: pancreatic adenocarcinoma
PPI: protein–protein interaction
TAM: tumor-associated macrophage
TCGA: The Cancer Genome Atlas
TIC: tumor-infiltrating immune cell
TME: tumor microenvironment
Treg: regulatory T cell
